# Potentials of organic manure and potassium forms on maize (*Zea mays* L.) growth and production

**DOI:** 10.1038/s41598-020-65749-9

**Published:** 2020-05-29

**Authors:** Essam E. Kandil, Nader R. Abdelsalam, Mansour A. Mansour, Hayssam M. Ali, Manzer H. Siddiqui

**Affiliations:** 1 Plant Production Department, Faculty of Agriculture, (Saba Basha), Alexandria University, Alexandria, 21531 Egypt; 2 Agricultural Botany Department, Faculty of Agriculture, (Saba Basha), Alexandria University, Alexandria, 21531 Egypt; 3 Zahra Higher Institute of Sciences and Technology, Tripoli, Libya; 4 Botany and Microbiology Department, College of Science, King Saud University, P.O. Box 2455, Riyadh, 11451 Saudi Arabia; 5 Timber Trees Research Department, Sabahia Horticulture Research Station, Horticulture Research Institute, Agriculture Research Center, Alexandria, 21526 Egypt

**Keywords:** Zea mays, Agroecology

## Abstract

Worldwide, maize (*Zea mays* L.) is considered an important food and fodder crop. Compost as a soil amendment and potassium (K) could enhance the maize yield. Therefore, two field experiments were carried out in the two seasons 2017 and 2018 to study the effects of compost at three levels and four forms of potassium fertilization on the yellow maize hybrid ‘*Pioneer* SC *30N11*’ yield components. To conduct the field trials, a split plot system in three replications was established. Three compost levels (0, 5 and 10 ton/ha) were in the main plots, and four potassium forms (untreated, nano-potassium fertilizer, humic acid and potassium sulfate) were in the subplots. Plot size was 10.50 m^2^, with 5 ridges with 3 m length and 0.7 m width. The results indicated that the application of compost (as organic manure) and the potassium forms significantly affected the plant height, ear length, grains number/rows, grains number/ear, 100- grain weight, straw and biological yields, grain protein and K contents in both seasons. Increasing the compost from 5 to 10 ton/ha increased the yield, its components, protein and K contents. The foliar application of nano-potassium followed by humic acid increased all the studied characteristics. The interaction between compost manure (10 ton/ha) and nano-potassium (500 cm^3^/ha) or humic acid (10 ton/ha) recorded the highest mean values for all parameters during both harvest seasons.

## Introduction

Maize is considered one of the three most vital cereal crops worldwide, come after wheat and rice. Maize is consumed as food and feed and used in several industries. The cultivated area in Egypt occupies approximately 935778 ha, producing up to 7.10 million tons of grains with an average yield of 7.60 ton/ha^1^.

Potassium fertilization plays a significant role in enhancing the yield, water use efficiency and nutrients use by maize plants. All recoverable soil potassium pools greatly affect the potassium availability, uptake and yield of maize that receives suitable irrigation, while water soluble and exchangeable potassium have developed as significant factors influencing the yields and potassium uptake by corn that is exposed to moisture stress when irrigation is inadequate^2^. In this respect, it is known that potassium has a main role in photosynthesis, water storage control and stomata opening in leaves^3^. Potassium deficiency significantly reduces the number of leaves and leaf size and, as a result, affects the photosynthetic activity of the plant^4^. Grain yields increase with increased potassium uptake under arid conditions^5^. Modern corn hybrid respond to potassium differently due to variations in their uptake, growth and utilization^6^. Potassium element is the highly abundant cation in plants and act main role in osmotic modification. When plant water capacity declines under drought condition, K can affect plants to accumulate solutes, lower their osmotic potential and enhance water inflow to maintain turgor pressure^7^. The absorption of sufficient amounts of potassium before drought helps plants maintain growth under abiotic 60 stress^8^.

Compost considers an effective element management strategy for keeping nitrogen (N) uptake and maize yields, reducing nitrogen loss and enhancing soil fertility. A considerable development in invertase activity in compost as organic fertilizer highlights the critical position of integrating the control of carbon and nitrogen for the sustainability of highly intensive agricultural production^9^. Furthermore, compost is an excellent soil amendment that adds a balance of nutrients while contributing valuable organic material to the soil. It has been well recognised that the incorporation of organic fertilizer into soil is more and more vital, as this practice increases soil fertility and crop yields^10^. The soil application of compost manure has a positive impact on basic soil characters. The composition of input substrates has a major effect on compost quality^11^. Organic fertilizers such as biofertilizers and humic substances are used to reduce the risks of salts, and the interaction between salinity and mineral fertilization with nitrogen and potassium^12,13^. Different works towards method innovations in soil fertility managing and incentive development for crop straw combined with natural fertilizer applications are required^14^. The application of compost manure significantly improved the grain yield of maize^15^. Combination with mineral and organic fertilization increased yield as compared with using mineral fertilization alone^16^. Maize growth and yield more than doubled over two crop cycles when compost manure and biochar were applied in combination on a calcareous soil^17^. There were positive responses of growth and yield of maize to the application of organic amendments, with smaller differences found between different organic amendments. Moreover, compost treatment significantly increased grain yield comparing with the control treatments^18^.

Application of humic acid (HA) resulted in high yields by developing physical and chemical characteristics of soil^19^. Furthermore, humic acid can significantly reduce water evaporation, increase yield/yield components, enhances the water retention and increases the water holding capacity in soil^20^. The addition of humic acid at 14.4 kg/ha was increased growth and grain yield of maize under water stress^21^. Application of humic substance (HS) significantly increased the soil fertility and micronutrient uptake^22^. Additionally, Hatami^23^ reported that applications of zinc and humic acid enhanced the yield under drought stress conditions, and cation exchange capacity of soil was significantly maximum when 100% NPK was used in combination with biochar at 7.5 ton /ha and humic acid. Humic acid caused a significant increase in organic carbon when applied at 7.5 t/ha^24^.

Nano-fertilizers postpone the release of nutrients and extended the period of fertilizer effects. Obviously, there was an opportunity for nanotechnology to significantly affect energy and the environment by enhancing fertilizers^25,26^. Applying nanoparticles (NPs) to plants is beneficial for plant growth and development due to their relatively greater absorbance and high reactivity^27^. Using nano-fertilizers as foliar applications at vegetative, flowering or grain- filling stage increased the yield and yield components^21,28^. A soil application of mineral fertilizer with a foliar application of nano- fertilizer showed the highest values for plant height, ear length, number of rows/ear, grains number/row, grains number/ear, 100- grain weight, and biological, straw and grain yields. Fertilizing the maize hybrid SC 168 with a foliar application of nano- fertilizer (K and P) and a soil application of mineral fertilizer (K and P) increased maize yield^29^. The plant height yield and yield components of wheat crop increased after the application of nano-fertilizer^30^. The application of nano-fertilizer promoted growth, development and antioxidant activity in sugar beet plants and improved crop production and plant nutrition. Moreover, nano-fertilizers have a great effect on the soil and can reduce fertilizer application frequencies^31^. Using nanoparticles with NPK nutrients increased the yield and its components of wheat compared with fertilization of mineral NPK in both seasons^19,32,33^ also^26^ indicated that using nanofertilizer increased the yield and quality characters of onion plants.

This investigation aimed to study the response of maize to compost and different potassium sources in addition study the gross income, net profit and cost benefit ratio of maize.

## Materials and methods

### Experimental setup

Two field experiments were carried out at Al- Huriya village, north of the El- Tahrir region, El- Behira Governorate, Egypt, during the seasons of 2017 & 2018 to study the effect of three levels (0, 5 and 10 ton/ha) of compost and four forms of potassium fertilization (control, nano- potassium fertilizer, HA and potassium sulfate) on the yield and yield components of a maize hybrid (*Pioneer* SC *30N11)*. The preceding filed crop was Berseem (Egyptian clover) during both seasons in the study. Some physical and chemical soil properties in the layer (0 to 30 cm depth) at the experimental site were analysed before sowing according to the method described by^34^; the summary can be found in Table (1). The analyses were conducted at the Soil Chemistry and Water Department, the Faculty of Agriculture, Saba Basha. The planting date was 10 May in the 2017 and 2018 seasons. The field was hand-thinned before the first irrigation to one plant per hill.

**Table 1:** Physical and chemical properties of the experimental soil.

Soil characteristics	Seasons	
	2017	2018
**Physical properties**		
Soil texture	Sandy loam	Sandy loam
Sand (%)	60.90	61.03
Silt (%)	10.60	10.05
Clay (%)	28.50	28.92
**Chemical properties**		
**(A) Soil Salinity and Sodicity**		
pH (1: 2.5 water suspension)	8.10	7.99
EC (dSm^−1^)	3.41	3.53
**(B) Soluble cations (mEq/L)**		
Ca^++^	7.60	8.00
Mg^++^	4.20	4.85
Na^+^	5.10	5.00
K^+^	0.50	0.55
**(C) Soluble anions (mEq/L)**		
HCO_3_^−^	3.00	3.95
Cl^−^	3.80	3.10
SO_4_^**−**^	10.30	10.20
O.M. (%)	1.85	1.90
CaCO_3_ (%)	22.50	23.70
**(D) Soil nutrients content (mg/kg)**		
Available mineral N (mg/kg)	22.40	25.60
Available P (mg/kg)	5.12	5.50
Available K (mg/kg)	2.10	1.90

### Experimental design

A split plot design with three replications was used. The three compost levels (0, 5 and 10 ton/ha) (Table 2) were assigned in the main plots, and the four forms of potassium fertilization, (untreated, nano-potassium fertilizer, humic acid and potassium sulfate) were allocated in the sub-plots. Each sub-plot consisted of 5 ridges with 3 m length and 70 cm in width, and the plot area was 10.5 m^2^ in both seasons.

**Table 2:** Composition of compost used in current study.

Determination	Compost
Moisture (%)	10.70
Organic matter (%)	45.30
Total N (%)	1.90
Total P (%)	1.70
Total K (%)	1.99
pH	6.53
EC	1.40
Fe (ppm)	2660
Zn (ppm)	55.00
Mn (ppm)	28.00
Cu (ppm)	12.50

Contents are available on the state (Bio Nano Technology Company, Egypt).

### Application of fertilizers

**Figure 1:**
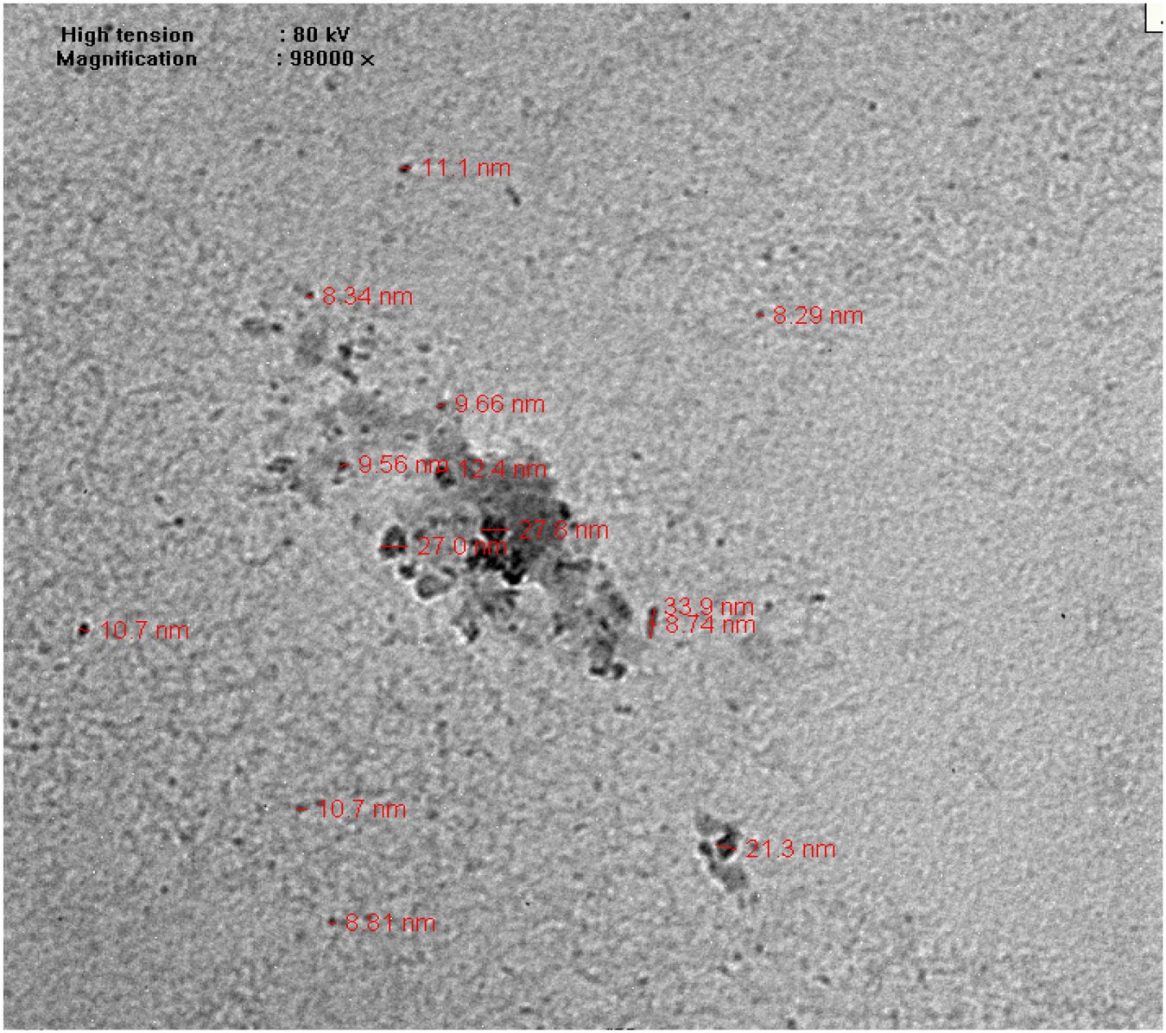
Transmission electron micrograph of Potacrystal (KNPs), the size ranged from 3.90–8.34 nm.

Potassium sulfate (K_2_SO_4_) (120 kg/ha) and humic acid from K- humate (10 kg/ha) were applied at sowing time during both seasons. Phosphorus fertilizer (60 kg P_2_O_5_/ha) was added before sowing in the form of (15.5% P_2_O_5,_ calcium super phosphate). Ammonium nitrate (NH_4_NO_3_ – 33.50 N %) (288 kg N/ha) was used as the nitrogen source and was applied equally on two doses. The first dose was applied before the first irrigation, and the second dose was added before the second irrigation during both seasons. A nano-compound (Potacrystal, 500 cm/500 litter water/ha) was used as foliar application twice, (45 and 65 days after sowing) (Table 3). The Potacrystal K NPs morphology was confirmed with a transmission electron microscope (TEM, Hitachi, Japan) at the Electron Microscopic Unit, Faculty of Science, Alexandria University. The shape and size of the Potacrystal K NPs were determined from the obtained TEM micrographs^35^ and are shown in Fig. (1).

**Table 3:** Structure of K NPs fertilizer used in current study.

Element	Nano-potassium fertilizer (Potacrystal)
K_2_O	36.0%
Amino acids	5.0%
Vitamins	1.0%
Total nitrogen	5.0%
Micronutrients (Br, Zn, Mn, Co and Mo)	2.0%

Contents are available on the state (Bio Nano Technology Company, Egypt).

### Data collection

At harvest, the three inner rows were used for the grain yield estimation. The following data were recorded:

#### Yield and its components

Plant height (cm), ear length (cm), grains number/rows, grains number/ear, 100-grain weight (g), grain yield (ton/ha), straw yield (ton/ha) and biological yield (ton/ha) were measured. Where, biological yield (ton/ha) = Grain yield + straw yield.

#### Grain characteristics

##### The grain protein content

Was calculated as the following formula Protein content (%) = Total nitrogen (%) x 6.25 to obtain the grain protein content (%) according to the method described by the Association of Official Analytical Chemists^36^:

##### The grain K content

Was determined by the vanadomolybdate yellow method as described by^37^, and the colour intensity that developed was read in a spectrophotometer at 405 nm.

##### Economic attributes

Economic analysis for current results were used to determine the variances between different factors levels to obtaining the greatest profitability of compost levels and potassium forms as compared with control. In the economic analysis, some economic criteria such as production costs, gross income, net profit and benefit cost ratio (BCR) were included. Economic criteria were estimated from the following formulas: gross income = grain yield (t) plus straw yield (t); net profit = gross income – production costs and benefit costs ratio (BCR) = $$\frac{{\rm{Gros}}\,{\rm{income}}}{{\rm{Cost}}\,{\rm{of}}\,{\rm{cultivation}}}$$ . It was calculated based on the basis of local market price prevailing the harvest time of the produce, the price of maize grain yield was in range from 231.01 to 243.59 $/t and the straw yield (silage) was in range from 24.05 to 25.64 $/t (Dollar exchange (1$) was = 15.80 and 15.60 L.E. in both seasons, respectively). The production costs were calculated in Egyptian pounds at the local market price then transferred to dollars ($) (see Table 4).

**Table 4:** Itemization of maize produces price during 2017 and 2018 seasons.

Items	Seasons and cost ($)	
	2017	2018
**A-Agricultural Input:**		
Land rent/ha for 4 months.	319.15	461.54
Ploughing and tillage/ha	37.97	46.15
Bund former and alignment channel costs/ha	25.95	28.85
Hoeing and herbicides of weeds/ha	85.44	96.15
**Fertilizer Price:**		
Phosphorus fertilizer (60 kg P_2_O_5_/ha)	49.05	50.0
Potassium sulphate (48-52% K_2_O) 120 kg/ha	31.65	38.46
HA from K- humate (10 kg/ha)	31.65	38.46
Ammonium nitrate (NH_4_NO_3_ – 33.50 N %) (288 kg N/ha)	217.72	230.77
K- NPs (0.5 L/ha)	2.50	2.50
Irrigation cost/ha	45.57	48.07
Harvesting cost/ha	37.97	44.87
**Grains price**	18.82	19.23
**Total cost**	903.44	1105.05
**B-produces price:**		
Grain yield of maize (t)	231.01	243.59
Green foliage of maize = straw yield (t)	24.05	25.64

### Statistics analysis

Analysis of variance was used according^38^. All statistical analyses were done by the CoStat computer software package^39^ CoStat (2005). The least significant difference test (LSD) at the 0.05 level of probability was used to compare the treatment means. Pearson’s correlation was used to analyze the relationship between yield parameters and soil amendments.

## Results and discussion

The results, as shown in Tables (5) and (6), indicated that the plant height, yield and its components during both seasons were significantly (*p* ≤ 0.05) affected by the compost levels, potassium sources and their interaction in the 2017 and 2018 seasons.

**Table 5:** Average values of plant height, ear length, grains number/row, grains number/row and 100- grain weight of maize hybrid “Pioneer SC-30N11” as affected by compost, the potassium fertilization forms and their interaction during two seasons (2017 and 2018).

Attributes	K- fertilizer sources (K)		2017 season							2018 season					
		Compost (ton/ha): (C)			Average (K)	LSD at 0.05			Compost (ton/ha): (C)			Average (K)	LSD at 0.05		
		0	5	10		K	C	K x C	0	5	10		K	C	K x C
Plant height (cm)	Untreated (No- K)	164.3	181.8	193.4	179.8b	6.6	5.7	11.4	173.3	189.8	201.4	188.2b	7.3	6.3	12.7
	Nano-potassium (K)	179.7	205.7	199.7	195.0 a				185.3	212.0	208.3	201.9a			
	Humic acid (HA)	174.0	203.7	195.7	191.1 a				182.0	209.3	203.6	198.3a			
	Potassium sulfate (K_2_SO_4_)	173.9	193.4	207.7	191.7 a				182.9	201.4	215.7	200.0a			
Average (C)		173.0 b	196.2a	199.2a					180.9b	203.1a	207.3a				
Ear length (cm)	Untreated (No- K)	16.0	16.9	18.3	17.1b	0.83	0.72	1.44	16.0	16.7	18.0	16.9c	0.73	0.64	1.7
	Nano-potassium (K)	16.0	19.3	19.3	18.2a				16.3	20.1	19.4	18.6a			
	Humic acid (HA)	17.3	17.6	18.4	17.8ab				16.4	18.6	18.0	17.7b			
	Potassium sulfate (K_2_SO_4_)	17.0	18.1	18.3	17.8ab				18.0	19.0	17.9	18.3ab			
Average (C)		16.6b	18.0a	18.6a					16.7b	18.6a	18.3a				
Grains number/row	Untreated (No- K)	32.0	39.3	41.0	37.4b	2.0	1.7	3.3	31.3	42.1	44.9	39.4ab	2.2	1.9	3.8
	Nano-potassium (K)	36.3	41.3	41.3	39.6 a				39.1	44.1	41.3	41.5a			
	Humic acid (HA)	34.3	37.7	43.3	38.4ab				37.1	39.9	44.9	40.6a			
	Potassium sulfate (K_2_SO_4_)	32.3	36.3	42.3	37.0b				33.9	39.2	41.7	38.3b			
Average (C)		33.7c	38.7b	42.0a					35.4b	41.3a	43.2a				
Grains number/ear	Untreated (No- K)	448.0	578.0	574.0	533.3bc				437.7	619.1	628.1	561.6bc			
	Nano-potassium (K)	508.7	604.0	661.3	591.3a	30.5	26.4	52.8	547.9	645.1	660.3	617.8 a	33.8	29.3	58.6
	Humic acid (HA)	480.7	527.3	663.3	557.1b				519.9	558.1	686.0	588.0ab			
	Potassium sulfate (K_2_SO_4_)	452.7	532.0	592.7	525.8c				474.1	574.0	583.3	543.8c			
Average (C)		472.5c	560.3b	622.8a					494.9c	599.1b	639.4a				
100- grain weight (g)	Untreated (No- K)	36.3	43.5	44.3	41.4b				35.3	42.3	43.3	40.3 c			
	Nano-potassium (K)	38.0	48.4	49.4	45.3a	2.2	1.9	3.8	40.7	44.0	49.9	44.9 a	2.4	2.1	4.2
	Humic acid (HA)	41.3	46.5	44.8	44.2a				38.3	47.0	48.3	44.5 ab			
	Potassium sulfate (K_2_SO_4_)	36.7	45.0	45.0	42.2b				37.9	43.1	46.5	42.5 bc			
Average (C)		38.1b	45.9a	45.9a					38.1 c	44.1 b	47.0 a				

Mean values in the same column/row marked with the same letters are not significantly different at the 0.05 level of probability.

ns.; not significantly difference at the 0.05 level of probability according to the LSD.

**Table 6:** Average values of biological yield, straw yield, grain yield, protein content and potassium content (K) of maize hybrid “Pioneer SC-30N11” as affected by compost, the potassium fertilization forms and their interaction during two seasons (2017 and 2018).

Attributes	K- fertilizer sources (K)		2017 season							2018 season					
		Compost (ton/ha): (C)			Average (K)	LSD at 0.05			Compost (ton/ha): (C)			Average (K)	LSD at 0.05		
		0	5	10		K	C	K × C	0	5	10		K	C	K × C
Biological yield (ton/ha)	Untreated (No- K)	13.2	15.0	15.9	14.7d	0.18	0.59	1.2	13.3	14.7	15.3	14.4c	0.19	0.52	1.0
	Nano-potassium (K)	14.7	16.7	16.9	16.1a				14.7	16.7	16.7	16.0a			
	Humic acid (HA)	14.4	16.3	16.5	15.7b				14.1	16.4	17.3	15.9a			
	Potassium sulfate (K_2_SO_4_)	14.7	16.5	15.4	15.5c				14.8	16.5	15.6	15.6b			
Average (C)		14.3b	16.1a	16.2a					14.2b	16.1a	16.3a				
Straw yield (ton/ha)	Untreated (No- K)	8.1	9.0	9.3	8.8 c	0.22	0.36	0.72	8.2	8.8	9.1	8.7c	0.33	0.37	0.74
	Nano-potassium (K)	8.7	9.5	9.9	9.4 a				8.7	9.8	9.9	9.5a			
	Humic acid (HA)	8.4	9.4	9.7	9.2 b				8.0	9.6	10.1	9.2a			
	Potassium sulfate (K_2_SO_4_)	8.5	9.7	9.0	9.1 b				8.6	9.8	9.1	9.1b			
Average (C)		8.4b	9.4a	9.5a					8.4b	9.5a	9.6a				
Grain yield (ton/ha)	Untreated (No- K)	5.1	6.0	6.6	5.9 c	0.14	0.39	0.78	5.1	5.9	6.2	5.7 c	0.26	0.31	0.62
	Nano-potassium (K)	6.0	7.2	7.0	6.7 a				6.0	6.9	6.8	6.6 a			
	Humic acid (HA)	6.0	6.9	6.8	6.6 a				6.1	6.8	7.3	6.8 a			
	Potassium sulfate (K_2_SO_4_)	6.2	6.8	6.4	6.5 b				6.2	6.7	6.4	6.4 b			
Average (C)		5.8 b	6.7 a	6.7 a					5.9 b	6.6 a	6.8 a				
Protein content (%)	Untreated (No- K)	6.7	7.9	8.9	7.8d				6.2	8.3	9.3	7.9c			
	Nano-potassium (K)	7.6	9.3	10.0	9.0a	0.38	0.47	0.18	7.6	9.2	9.6	8.8a	0.20	0.45	0.77
	Humic acid (HA)	7.5	8.5	9.3	8.4b				7.7	8.9	9.7	8.8a			
	Potassium sulfate (K_2_SO_4_)	7.1	8.7	8.3	8.0c				7.4	9.5	8.8	8.6b			
Average (C)		7.2c	8.6b	9.1a					7.2b	9.0a	9.4a				
K content (%)	Untreated (No- K)	1.2	1.1	1.3	1.2c				1.2	1.5	1.6	1.4c			
	Nano-potassium (K)	1.3	2.0	1.7	1.7a	0.13	0.15	0.25	1.3	2.1	2.0	1.8a	0.13	0.19	0.33
	Humic acid (HA)	1.4	1.2	1.8	1.5b				1.8	1.5	1.9	1.7a			
	Potassium sulfate (K_2_SO_4_)	1.1	1.3	1.6	1.3bc				1.4	1.6	1.6	1.5b			
Average (C)		1.3c	1.4b	1.6a					1.4b	1.7a	1.8a				

Mean values in the same column/row marked with the same letters are not significantly different at the 0.05 level of probability.

ns.; not significantly difference at the 0.05 level of probability according to the LSD.

### Effects of organic manure (compost) on the growth and yield of maize

The results as shown in Table (5), revealed that increasing compost rate from 0 up to 10 ton/ha increased plant height, ear length, grains number/row grains number/ear and 100- grain weight, furthermore, the lowest mean values of these traits were found in the control treatment during the two growing seasons. The results in Table (6) reported that the application of compost manure at either 5 or 10 ton/ha presented in the maximum biological yield, straw yield and grain yield. However, the grain protein content and grain K content differed and were affected significantly (*p* ≤ 0.05) by 10 tons/ha of compost. Application of compost at the rate of 10 ton/ha gave the highest mean values of yield characters of maize followed by 5 ton/ha of compost which gave the same results as compared with the control treatments in the two seasons. The lowest values of the plant attributes were obtained in the control treatment during both growing seasons. Using of organic manure into soil is more and more important, as this practice improves the soil fertility with increasing the crop yields^10^. These findings results agree with those recorded by^18,19,40^ who reported that compost (5 ton/ha) increased crop productivity in terms of the yield and yield component of maize. Compost is an effective nutrient management strategy to maintain N uptake and maize yields, decrease N loss and increase soil fertility^9–20^. Additionally, organic fertilizer modifications, such as green manure and crop straw return, have been widely recommended as practices for improving yields of crop, while increasing the soil quality^33,41,42^. Organic amendments can directly enhance crop growth by improving soil nutrient availability^42^.

Regarding to correlation between soil amendments in growth and yield characters, the results in Tables (7 and 8) revealed that there was strong positive correlation between soil amendment (compost) and plant height, ear length, grains number/row, grains number/row, 100- grain weight, biological yield, straw yield, grain yield, protein content, and potassium content (K) during 2017 and 2018 seasons. The results showed that the plant height, ear length, grains number/row, grains number/row, 100- grain weight, biological yield, straw yield, grain yield, protein content, and potassium content (K) are positively correlated with each other under application of soil amendments except the correlation between soil amendments and number of grains/ear which had no significant correlation with plant height and ear length. On the other hand, 100- grain weight had no significant correlation between number of grains/ear. This results could be due to the vital role of the compost application for improving soil properties and increasing availability of nutrients to maize plants. On the other study, compost treatment increased soil fertility and nutrient up take of plants which caused enhancing growth, yield and yield components^9,43^. The current study in a line with^44–46^ who pointed to the important of soil amendments in recent years in agriculture and their role in improve the soil quality and increase crop yield. Other studies for Doan *et al*.^47^, reported that there are significant differences in crop yields because of the organic amendments which can be clearly shown in water stress conditions, thus will increase the yield in crops and^48,49^ showed that there are correlation between the high application rates of organic amendments and better performance of maize yield.

**Table 7:** Pearson’s correlation coefficients (r ~ values) between soil amendments and plant height, ear length, grains number/row, grains number/row, and 100- grain weight during both seasons (2017 and 2018).

	Seasons	Soil amendments	Plant height (cm)		Ear lengt (cm)		No. grains/row		No. grains/ear		100- grain weight (g)	
			2017	2018	2017	2018	2017	2018	2017	2018	2017	2018
Soil amendments		1										
Plant height (cm)	2017	0.72**	1									
	2018	0.74**	0.99**	1								
Ear length (cm)	2017	0.64**	0.66**	0.67**	1							
	2018	0.49**	0.65**	0.64**	0.72**	1						
No. grains/row	2017	0.54**	0.60**	0.61**	0.55**	0.59**	1					
	2018	0.69**	0.75**	0.75**	0.78**	0.69**	0.77**	1				
No. grains/ear	2017	0.26 ns	0.33 ns	0.32 ns	0.31 ns	0.44**	0.57**	0.40**	1			
	2018	0.34*	0.54**	0.50**	0.42 ns	0.50**	0.52**	0.55**	0.77**	1		
100- grain weight (g)	2017	0.71**	0.74**	0.73**	0.63**	0.62**	0.57**	0.76**	0.26 ns	0.49**	1	
	2018	0.78**	0.69**	0.68**	0.63**	0.54**	0.57**	0.76**	0.27 ns	0.39	0.82**	1

**Table 8:** Pearson’s correlation coefficients (r ~ values) between soil amendments and biological yield, straw yield, grain yield, protein content, and potassium content (K) during both seasons (2017 and 2018).

	Seasons	Soil Amendments	Grain yield (t/ha)		Straw yield (t/ha)		Biological yield (t/ha)		Protein content (%)		K content (%)	
			2017	2018	2017	2018	2017	2018	2017	2018	2017	2018
Soil Amendments		1										
Grain yield (t/ha)	2017	0.52**	1									
	2018	0.52**	0.88**	1								
Straw yield (t/ha)	2017	0.66**	0.70**	0.70**	1							
	2018	0.62**	0.59**	0.71**	0.84**	1						
Biological yield (t/ha)	2017	0.64**	0.93**	0.86**	0.92**	0.77**	1					
	2018	0.62**	0.79**	0.91**	0.84**	0.94**	0.88**	1				
Protein content (%)	2017	0.76**	0.61**	0.65**	0.74**	0.73**	0.73**	0.75**	1			
	2018	0.79**	0.76**	0.77**	0.74**	0.71**	0.81**	0.80**	0.82**	1		
K content (%)	2017	0.53**	0.42**	0.54**	0.47**	0.53**	0.48**	0.58**	0.62**	0.51**	1	
	2018	0.48**	0.49**	0.58**	0.53**	0.52**	0.55**	0.59**	0.57**	0.58**	0.73**	1

### Effects of the potassium forms on the growth and yield of maize

Respecting to the potassium form effects on the plant height (cm), ear length (cm), grains number/row, grains number/ear, 100-grain weight (g), biological yield (ton/ha), straw yield (ton/ha), grain yield (ton/ha), protein content and K content (%) in both seasons are shown in Tables (5 and 6). The results in Table (5) demonstrate that, the potassium forms significantly influenced (*p* ≤ 0.05) the maize yield and their components compared to control. Generally, nano-potassium significantly (*p* ≤ 0.05) affected the numerous tested attributes during two seasons. However, the lowest values were recorded in the treatment without amendments (No- K) during both seasons. These results agree with those obtained by^19,27,29,31,50^, they indicated that nano- particles applications to plants had beneficial effects on the growth and yield for most tested crops. On the other hand,^15,19,20,23^ revealed that applied HA levels significantly increased yield of examined crops.

Regarding the potassium forms, as shown in Table (6), significant (*p* ≤ 0.05) effects on the biological, straw and grain yield; protein and K contents were found during the two growing seasons. The highest biological and straw yield (ton/ha) were recorded with nano-potassium in the 1^st^ season, while in the in the 2^nd^ season were with nano-potassium (K NPs) and HA. However, the highest grain protein and K contents were observed with the foliar applications of nano-potassium in the 2017 and 2018 seasons, respectively, while the lowest percentages were recorded in the treatments with no amendments (No- K) in both seasons. Increasing of the grain yield due to the increase in the yield components of maize. These findings are in the same line with those obtained by^27,31^, who indicated that applications of K NPs on plants had positive and supportive functions for the growth and yield of crops. Nano- potassium (K NPs) increased yield followed by application of HA under the study conditions. On the other hand, application of humic acid (HA) enhanced growth, yield and yield components and protein content (%) of maize, in addition, application of humic acid be able to as a growth regulator, increasing stress tolerance, improving soil characteristics and enhancing nutrients availability^51–55^.

### Effect of the interaction between organic manure (compost) and potassium forms on growth and yield of maize

The results found in Table (5) showed that the interaction between compost and the forms of potassium significantly affected growth and yield characters during the two seasons. The application of compost at the rate of 10 ton/ha plus potassium sulfate resulted in the tallest plants and heaviest 100-grain weight; however, compost plus HA resulted in the highest grains number/row and grains number/ear in the two seasons. Moreover, the lowest values of this traits were obtained in the untreated plants for the two studied independent variables during both seasons. The data in Table (6) shows the interaction between compost and the forms of potassium significantly affected the maize yield and grain characteristics (protein and K contents) during both seasons. The application of compost at 10 ton/ha with nano-potassium (K NPs) resulted in the largest biological yield, straw yield (ton/ha) and protein content in the 1^st^ season 2017 and the highest K content in the 2^nd^ season 2018. However, in the second seasons, the largest biological yield (ton/ha), straw yield and protein content were recorded with the application of 10 ton/ha of compost and a foliar application of HA. On the other hand, the highest K content (2.0%) was obtained with the application of compost at 5 ton/ha and nano-K in 2017 season. The heaviest grain yield (7.2 ton/ha) was recorded with 5 ton/ha of compost plus nano-K fertilizer in the first season. However, in the second season 2018, the maximum grain yield (7.3 ton/ha) was obtained with the application of 10 ton/ha of compost with HA. Moreover, the minimum values were obtained in the control treatment during both seasons. Results showed that there is significant interaction between the compost (ton/ha) and potassium forms in both seasons.

The increase of grain yield of maize crop may be due to mainly it was attributed to plant height, ear length, number of grains/ear, number of rows/ear, and 100- grain weight. Amongst the treatments, the application of compost, K NPs and HA recorded the highest plant height, ear length, number of grains/ear, number of rows/ear, and 100- grain weight. Based on other study, the application of bio-organic fertilizer improved soil fertility as well as the growth and yield characters^56^. In that context, combination of organic manure with inorganic fertilizer resulted in a high growth and yield of maize and wheat^9,57^. On the other hand, organic fertilizer increases yield if it is applied to provide additional nutrients based on inorganic fertilizer application i.e. NPK^58,59^. Additionally^60^ revealed that NPK fertilizers plus organic manure enhanced crop yields higher than NPK fertilizers alone or NPK fertilizers plus straw. However^61^, showed that using NPK NPs with organic manure increased growth, yield of maize. In addition, combination between foliar application KP NP_S_ with soil application of mineral NP increased growth and yield of maize^29^.

### Effects of compost and the potassium forms and their interaction on the gross income, net profit and benefit- cost ratio (BCR) of maize

Gross income is an important economic index that determines the profit or benefit that a farmer can obtain. On the other hand, net return reflects the actual income of farmer. While benefit cost ratio (BCR) is an index that shows the comparative explanation about the investment by a farmer. The results in Table (9) revealed that there was a significant difference in gross income, net profit and benefit cost ratio among tested treatments. The results revealed that increasing compost rates from 5 to 10 ton/ha increased gross income, net profit and BCR as comparing with control treatments (untreated). Also, using K forms increased gross income, net profit and BCR as comparing with control treatments (untreated). Where the highest values were obtained with application of 5-ton compost/ha, while least value was given by control (0 compost). Also, using nano K or humic acid achieved the highest values, while the lowest one recorded with control (No- K) during the two seasons. Application of compost at the rate of 5 ton/ha with humic acid recorded the highest values of gross income, net profit and benefit cost ratio, meanwhile the lowest ones gave with control treatment in the two seasons (Table 9). These results are in harmony with those obtained by^62–64^ which showed that incorporation of soil fertility improvement measures make soil moisture conservation more profitable. On the other hand, production of healthy and good quality crop will help the farmers to maximize net profit based on gross income^65^.

**Table 9:** Economics of the maize hybrid “Pioneer SC-30N11” as affected by compost, the potassium fertilization forms and their interaction during both seasons.

Attributes	K- fertilizer sources (K)		2017 season						2018 season						
		Compost (ton/ha): (C)			Average (K)	LSD at 0.05			Compost (ton/ha): (C)			Average (K)	LSD at 0.05		
		0	5	10		K	C	K x C	0	5	10		K	C	K x C
Gross income ($)	Untreated (No- K)	1383.2	1615.8	1742.0	1580.3b	85.6	205.3	148.3	1457.0	1656.7	1743.4	1619.0b	83.8	127.3	145.1
	Nano-potassium (K)	1593.4	1845.7	1818.7	1752.6a				1679.7	1951.4	1925.1	1852.1a			
	Humic acid (HA)	1597.2	1892.1	1872.5	1787.3a				1695.7	1910.1	2014.2	1873.3a			
	Potassium sulfate (K_2_SO_4_)	1639.2	1808.0	1690.6	1712.6a				1726.5	1889.1	1782.8	1799.5a			
Average (C)		1553.3b	1790.4a	1781.0a					1639.7b	1851.8a	1866.4a				
Net profit ($)	Untreated (No- K)	479.7	712.4	838.6	676.9 b	85.7	205.4	148.4	352.1	551.7	638.4	514.1b	83.8	127.3	145.2
	Nano-potassium (K)	689.9	942.2	915.3	849.1 a				574.7	846.4	820.1	747.1a			
	Humic acid (HA)	693.7	988.6	969.1	883.8 a				590.7	805.1	909.2	768.3a			
	Potassium sulfate (K_2_SO_4_)	735.8	904.6	787.2	809.2 a				621.5	784.1	677.8	694.5a			
Average (C)		649.8 b	887.0a	877.6a					534.8b	746.8a	761.4a				
Benefit costs ratio (BCR)	Untreated (No- K)	1.5	1.8	1.9	1.7b	0.1	0.2	0.2	1.3	1.5	1.6	1.5b	0.1	0.1	0.2
	Nano-potassium (K)	1.8	2.0	2.0	1.9a				1.5	1.8	1.7	1.7a			
	Humic acid (HA)	1.8	2.1	2.1	2.0a				1.5	1.7	1.8	1.7a			
	Potassium sulfate (K_2_SO_4_)	1.8	2.0	1.9	1.9a				1.6	1.7	1.6	1.6a			
Average (C)		1.7b	2.0a	2.0a					1.5b	1.7a	1.7a				

Mean values in the same column/row marked with the same letters are not significantly different at the 0.05 level of probability.

## Conclusion

On average, grain yield and quality of maize crop was significantly increased by application of compost, K- forms and their interactions. The highest increase was found with application of compost at the rate of 5 or 10 ton/ha with K NPs or humic acid (HA) which resulted in maximum plant height, ear length, 100- grain weight, grain yield, straw yield, biological yield, grain protein concentration (%) and grain K content (%) of maize varieties. However, these treatments recorded the highest gross income, net profit and benefit costs ratio (BCR). Therefore, using combination with compost (5 ton/ha) and K NPs (500 cm/ha) or humic acid (10 kg/ha) for getting higher yield, its components and quality of maize crop under the study conditions. With this combination, it will be possible for farmer to improve the growth, yield and quality of maize hybrid ‘Pioneer SC 30N11’ with the gross income, net profit and benefit costs ratio (BCR). Therefore, the best treatments were 5 ton/ha of compost + 0.5 l/ha of K NPs or + 10 kg/ha of HA which achieved the highest net profit under the study conditions. However, additional study is needed to investigate the effect of higher levels of K- NPs with humic acids and compost manure as soil amendments on maize yield and quality and its effects on soil properties and elements availability.
